# The Association between the Respiratory System and Upper Limb Strength in Males with Duchenne Muscular Dystrophy: A New Field for Intervention?

**DOI:** 10.3390/ijerph192315675

**Published:** 2022-11-25

**Authors:** Agnieszka Sobierajska-Rek, Eliza Wasilewska, Karolina Śledzińska, Joanna Jabłońska-Brudło, Sylwia Małgorzewicz, Andrzej Wasilewski, Dominika Szalewska

**Affiliations:** 1 Department of Rehabilitation Medicine, Faculty of Health Sciences with the Institute of Maritime and Tropical Medicine, Medical University of Gdansk, 80-219 Gdańsk, Poland; 2 Department of Pulmonology and Allergology, Faculty of Medicine, Medical University of Gdansk, 80-211 Gdańsk, Poland; 3 Department of Internal and Pediatric Nursing, Faculty of Health Sciences with the Institute of Maritime and Tropical Medicine, Medical University of Gdansk, 80-211 Gdańsk, Poland; 4 Department of Clinical Nutrition, Faculty of Health Sciences with the Institute of Maritime and Tropical Medicine, Medical University of Gdansk, 80-416 Gdańsk, Poland; 5 Student Scientific Association at Department of Physical Education and Sport, Wroclaw Medical University, 51-601 Wroclaw, Poland

**Keywords:** Duchenne muscular dystrophy, pulmonary function, pulmonary function test, MIP, MEP, upper limbs, respiratory muscles, hand grip, hand grip strength, PUL

## Abstract

Progressive, irreversible muscle weakness is the leading symptom of Duchenne muscular dystrophy (DMD), often resulting in death from respiratory muscle failure. Little is known about the relationship between the functioning of the respiratory system and the hand grip—a function which remains long preserved. This study aimed to investigate the interdependence between muscle strength and the function of both hand grip and the respiratory system in patients with DMD. Materials and Method: The study included cohort patients, aged 6–17, with DMD, recruited from the Rare Disease Centre, Gdansk, Poland. Clinical status (Vignos scale, Brook scale), pulmonary function (respiratory muscle strength—MIP, MEP); spirometry (FEV1; FVC), as well as upper limb function (performance of the upper limb—PUL 2.0) and hand grip strength (HGS) (hand-held dynamometer) were evaluated in all participants. Results: Finally, 53 boys (mean age 11.41 ± 3.70 years, 25 non-ambulant) were included. Each of the participants presented a lower %pv of MIP (48.11 ± 27), MEP (38.11 ± 22), PUL (75.64 ± 27), and HGS (33.28 ± 18). There were differences between the ambulatory and non-ambulatory groups in values of MIP, MEP, FVC, PUL, HGS (*p* < 0.001 for all), and FEV1 (*p* < 0.013). There were correlations between PUL, HGS, and MIP (R = 0.56; R = 0.61, *p* < 0.001 both), MEP (R = 0.59; R = 0.62, *p* < 0.001), FVC (R = 0.77; R = 0.77, *p* < 0.001), and FEV1 (R = 0.77; R = 0.79; *p* < 0.001). These correlations were found for all participants, but non-ambulatory patients presented stronger relationships. Conclusions: 1. The pulmonary and upper limb functions were within the normal range in ambulatory and low in non-ambulatory patients with DMD, but the muscle strength of both systems was low, regardless of the stage of the disease. 2. There seems to be an interdependence between the respiratory system and upper limb strength in terms of muscle strength and function in DMD patients, which is stronger in non-ambulatory patients. This may be the basis for the creation of a new personalized plan in rehabilitation—the simultaneous rehabilitation of the respiratory and upper limb muscles. Further studies on this theory should be conducted.

## 1. Introduction

In progressive irreversible neuromuscular disorders(DMD; OMIM Online Mendelian Inheritance in Man 310200), mutations in the dystrophin gene lead to the impaired production of dysfunctional dystrophin protein, which impacts appropriate muscle activity [1]. Usually, the first DMD symptoms occur in early childhood, at 2 to 3 years old, when boys do not run as quickly as their peers, start to fall more often, and have difficulties climbing stairs. This is due to the progressive muscular weakness of the proximal muscles of lower limbs and pelvic girdle, with consecutive loss of ambulation, mostly in the teenage years.

Steady disease progression deteriorates respiratory muscle function, resulting in restrictive ventilator defect [2,3]. Due to respiratory muscle exhaustion, the lungs cannot expand fully, which causes decreased lung volume, inadequate ventilation, and insufficient oxygenation [4]. Consequently, the risk of developing hypoventilation, and the subsequent dependence on ventilatory support increases with age, usually after the loss of ambulation. Pulmonary function reflects the skeletal muscle condition in DMD patients. The force of respiratory muscles generates pressure fluctuations, and the reduction in maximal pressures indicates respiratory muscle fatigue. The pulmonary function test (spirometry) measures lung volumes (FVC, FEV1), while maximal respiratory pressure reflects respiratory muscle strength (maximal inspiratory pressure—MIP; maximal expiratory pressure—MEP). The restriction of diaphragm movements has been revealed during maximal inspiration in DMD patients with an MIP of less than 60% of the predicted value [5]. Another study showed that patients with an MEP less than 60 cmH2O are at risk of losing the ability to cough effectively [6]. Consequently, these changes lead to the insufficiency of the respiratory system, which is one of the leading cause of death in DMD patients, alongside cardiomyopathy [3,4,5,6,7].

Unlike the respiratory system, the upper limbs retain their strength and function longer than any other muscular system in patients with DMD. Particularly, the function of the distal muscles of the upper limb is usually retained longer, even at the advanced stage of the disease. It was found that hand strength within the first decade of life increases, and in the following years, significantly correlates with the degree of physical disability [7]. The milestone in disease progression for the upper extremities is the loss of ability to reach over the head, loss of ability to self-feed, loss of ability to place hands on a tabletop, and to use a computer (distal hand function) [8,9]. Such activity is particularly important for DMD patients, as it allows them to remain independent, at least to some extent, and helps to provide a satisfactory and meaningful life.

The close anatomical relationship between the muscles of the upper limbs and the respiratory muscles (for example, the muscles of the upper extremities and the fascia surrounding most dorsal muscles of the trunk) makes it possible for them to influence each other [10].

Although this interconnectedness is an important issue, there is little data regarding the comparison of upper limb capacity and respiratory system function in these patients, and the results are inconclusive [11,12]. Moreover, the data from our previous study showed that pulmonary dysfunction in children with DMD may appear earlier than thought [2], and some ambulatory patients may not be aware of the gradual lung volume decrease, because they do not experience symptoms of nocturnal hypoventilation [13]. The mutual influence of the respiratory system and the upper limbs in patients with DMD is still little described and understood.

Therefore, the aim of the study was to investigate the interdependence between muscle strength and function of both the respiratory system and the upper limbs in patients with DMD.

## 2. Materials and Methods

### 2.1. Study Design

This cross-sectional single-center study was conducted as a part of the Multidisciplinary Care Program for Patients with Duchenne Muscular Dystrophy at the Rare Disease Center (RDC), University Clinical Center, Medical University of Gdansk, Poland, within the years 2017–2021. The University Clinical Center is a member of the TREAT NMD Alliance Neuromuscular Network.

All patients and/or their legal guardians provided written informed consent prior to participation in the study. Approval for the study was obtained from The Committee of Ethics, no: NKBBN/611/2017/2018, NKBBN/260/2021, conforming to the principles embodied in the Declaration of Helsinki.

### 2.2. Patients and Methods

#### Participants

The study included cohort DMD patients, aged between 5–17 years, diagnosed based on the presence of clinical symptoms, genetic testing, and/or muscle biopsy result [1]; patients had the ability to maintain a sitting position in a wheelchair and to perform pulmonary function tests.

The exclusion criteria were: lack of written consent, continuous dependence on assisted ventilation, presence of respiratory tract infection, and severe behavioral or attention deficit disorders that prevented adequate cooperation during evaluation.

Clinical status was assessed using the Vignos (VS) [14] and the Brooke (BS) [15] scale. The anthropometric data—weight and height—with an accuracy of 0.1 kg and 0.5 cm respectively, were obtained; body mass index (BMI) was calculated as the weight divided by height squared (kg/m^2^). BMI Polish references were used to calculate z-scores [16]. The height of non-ambulant participants was calculated from the ulnar length [17]. Patients were qualified as non-ambulant when they were unable to walk more than ten meters without human assistance.

The evaluation of pulmonary status consisted of spirometry and maximal respiratory pressure assessments, conducted by the same pulmonologist experienced in patients with DMD. The function and strength of the upper limbs were assessed using the performance of the upper limb (PUL) test and the maximal grip strength hand-held dynamometer by the same trained physical therapist and specialist in rehabilitation medicine.

### 2.3. Pulmonary Status

#### 2.3.1. Respiratory Muscle Strength

Maximal respiratory pressure was evaluated using a calibrated portable hand-held mouth pressure meter (MicroRPM; Micro Medical Ltd., Rochester, England) in a sitting position. To measure maximal expiratory pressure (MEP cmH2O), participants were asked to exhale with a maximum expiratory effort for at least one second, done after maximum inspiration. On the contrary, maximal inspiratory pressure (MIP cmH2O) was measured as a maximum inspiratory effort for at least one second, done after maximum expiration. The highest positive MEP value and the lowest negative MIP value were chosen from three or more attempts (five attempts maximum). The percentage predicted value of MEP (MEP%) and MIP (MIP%) values were calculated according to the formulas presented in Appendix A.

#### 2.3.2. Spirometry

Spirometry was performed using the calibrated, computerized spirometer Pneumo Screen (Jaeger, Germany), according to the European Respiratory Society and American Thoracic Society recommendations [18,19]. A minimum of three, and up to five, maneuvers with maximum effort were attempted by each subject. The highest value of forced vital capacity (FVC), forced expiratory volume in one second (FEV1), expressed as liters (L), and the percentage of predicted value (%pv) were evaluated from the correct acceptable attempts.

### 2.4. Upper Limb

#### 2.4.1. Performance of the Upper Limb (PUL)

PUL is a functional scale designed specifically to assess upper limb function in DMD patients. It is used to assess the functioning of the upper limbs and is subdivided into three parts (shoulder level, mid-level, and distal level), related to the activities of daily life that both patients and clinicians identify as relevant. Participants were asked to lift the weight of 500 g and 1 kg by shoulder flexion, bring a 200 g cup to the mouth, bring two hands onto a table, and move weights of 100 g, 500 g, and 1 kg on the table to the level of the waist, move a heavy can diagonally on a table, stack 5 heavy cans, one on top of the other, remove the lid from a container, tear a piece of paper, trace a path with a pencil, push on a light, collect coins with a dominant hand from a table, and pick up 10 g with a finger pinch. All activities can be scored with 1 or 2 points. One point is given if the task is performed, with compensations. A total score of 42 points can be achieved by adding the results of the three levels, with a higher score denoting better function [20]. Percentage values were calculated based on the assumption that every healthy child in the age commensurate to that in study group would achieve 42 points (100%).

#### 2.4.2. Maximal Hand Grip Strength (HGS)

Maximal HGS was evaluated with a dynamometer FT-5988—N1 (Spais, Gdansk, Poland)—a high-precision electronic device developed to measure grip strength in both patients with neuromuscular disorders and healthy individuals. During the test, patients were seated on a chair or wheelchair, in front of a table, with the dominant forearm placed on the table at waist level. All patients were strongly encouraged by evaluators to provide maximal voluntary isometric contractions for 3 s, followed by a rest period of about 30 s. Patients were asked to perform three attempts; the mean value was then calculated. Percentage predicted values were calculated according to the predictive equation formula presented in Appendix A [18].

### 2.5. Statistical Analysis

The results of the statistical analysis were expressed as mean and standard deviation (SD) or median and interquartile range (IQR). Correlations between numerical parameters were analyzed using the Spearman correlation test. The Mann–Whitney U test for non-parametric conditions was performed to analyze the differences between the groups. Linear regression analysis was used for the multivariate analysis of numerical parameters; p values less than 0.05 were accepted as significant. The statistical analysis was carried out with Statistica 13.3 (StatSoft, Kraków, Poland).

## 3. Results

### 3.1. Participant Characteristics

Finally, the study cohort consisted of 53 boys and adolescents with DMD. The mean age was 11.41 ± 3.70 years; 25 patients were non-ambulant. The mean age at loss of ambulation was 10.41 ± 2.06 years. Ambulatory patients were younger than non-ambulatory patients (9.17 ± 2.85 vs. 13.92 ± 2.87; *p* < 0.001).

Ambulatory status assessed with VS varied from 1 to 9 points (median 3; IQR 8), and the general condition of the upper limbs with BS varied from 1 to 5 (median 2; IQR 2). There was relationship between age and ambulatory status (R = 0.65; *p* < 0.000). Detailed characteristics of the participants are presented in Table 1.

### 3.2. Respiratory System

**Figure 1:**
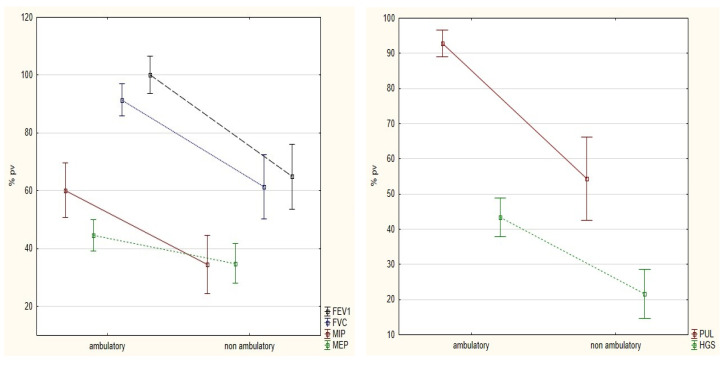
Differences in muscle strength and function of the respiratory system (**left**) and upper limbs (**right**) between ambulatory vs. non-ambulatory participants. Muscle strength (MIP, MEP, HGS) decreased in both ambulatory and non-ambulatory patients, while function indicators (FVC, FEV1, PUL) decreased only in non-ambulatory patients. %pv—%predicted value; FEV1—forced expiratory volume in 1 s; FVC—forced vital capacity; MIP—maximal inspiratory pressure; MEP—maximal expiratory pressure; PUL—performance of upper limb; HGS—hand grip strength.

The results of respiratory muscle strength and spirometry are shown in Table 2. The MIP ranged from 5 to 83 cmH2O and the MEP from 8 to 77 cmH2O. The participants presented lower values of MIP (mean 48.11 %pv) and MEP (mean 38.11 %pv) than normal for a healthy population [19]. There were differences between the ambulatory and non-ambulatory groups in MIP %pv and MEP %pv (*p* < 0.001 for both), and in spirometry parameters FVC %pv and FEV1 %pv (*p* < 0.001 and *p* < 0013, respectively) (see Figure 1).

### 3.3. The Upper Limb

The PUL scores for the assessed population ranged from 2 to 42 points, while the HGS ranged from 0.3 to 17.17 kg. There were differences between the ambulatory and non-ambulatory groups regarding PUL and HGS (92.85 %pv vs 54.38 %pv, and 43.35 %pv vs. 21.52 %pv, *p* < 0.001 for both); see Figure 1. None of the study groups achieved the results achieved by a healthy population of children and adolescents. Healthy young individuals always presented 100 %pv (after converting the nominal values according to age). Details of the upper limb assessment are presented in Table 3.

### 3.4. Associations between the Respiratory System and Muscle Function of the Upper Limb

Detailed data on associations between the respiratory and upper limb parameters are shown in Table 4. For the whole study population, there were correlations between PUL and respiratory muscle MIP, MEP (R = 0.44, *p* < 0.001; R = 0.54, *p* < 0.001, respectively), and spirometry parameters FVC, and FEV1 (R = 0.28, *p* = 0.04 and R = 0.35, *p* = 0.01, respectively). Moreover, there were correlations between hand grip strength and MIP and MEP (R = 0.38, *p* = 0.004, R = 0.45, *p* < 0.001, respectively), as well as FVC and FEV1 (R = 0.71, *p* < 0.001; R = 0.73, *p* < 0.001, respectively).

The univariable regression analysis showed that hand grip strength was related to PUL, FVC values, and age (see Table 5).

## 4. Discussion

In the present study, the relationship between the respiratory system and the upper limbs was evaluated in the cohort of 53 ambulant and non-ambulant Caucasian patients with DMD, aged 6–17 years.

The most important results of our study were (1) a strong correlation between the respiratory system (expressed as respiratory muscle strength and lung volume) with upper limb function and hand grip strength in patients with DMD, (2) the strongest correlation between the systems was observed in the non-ambulatory patients, (3) low values of muscle strength (MIP, MEP, HGS) were recorded in all participants, as opposed to low function (FEV1, FVC, PUL) recorded only in non-ambulatory patients, and (4) a high standard deviations for all measured parameters. These findings may indicate the relationship in terms of function and muscle strength between the respiratory system and strength of the upper limbs.

Studies by other authors showed comparable results [21,22]. Lee et al. evaluated 43 non-ambulatory Duchenne males, aged 10–30 years, and confirmed that their PUL scores correlate with their pulmonary function [23]. Additionally, in a cohort of 89 DMD boys, aged up to 18 years, the study of Ricotti et al. showed an association between respiratory and hand grip strength; moreover, the authors point to the possibility of the novel concept of a composite endpoint in clinical trials by combining respiratory function, upper limb strength, and force domains [11].

The nature of the association between hand grip strength and lung function remains unclear, although studies also confirm this relationship in patients with other diseases [24,25] and even in healthy persons [26,27]. For example, research on neuromuscular diseases, such as spinal muscular atrophy (SMA), showed that FVC% correlate positively with the upper limb function measured with the revised upper limb module (RULM) [28]. Moreover, research on sufferers from pulmonary diseases reported significantly decreased hand grip strength in children with asthma in comparison to healthy subjects [25]. Moreover, adults with chronic obstructive pulmonary disease and sarcopenia presented low hand grip strength associated with worse pulmonary function [24,29].

Interestingly, studies conducted on a healthy population of young [30] middle-aged adults [27] and healthy elderly [12,31] also indicate the relationship between hand grip strength and FEV1 and FVC. Even if the study examined only the muscle mass (evaluated by bioelectrical impedance) without hand grip strength, it also confirmed associations between pulmonary and upper limb function [26].

The next interesting result of our study showed that MIP, MEP, HGS, and PUL values were characterized by a high standard deviation. Meier et al. reported high coefficients of variability for MIP (18%) and MEP (15%), irrespective of age, in a group of 64 DMD patients, aged 10–18 years [32]. The cited study concluded that patients have difficulty regarding the reproducibility of the measurements.

In our center (CRD), studied children underwent training before pulmonary measurements were obtained. Despite this, we found a large dispersion in the results of the study group. According to Hogrel et al. significant variability of HGS in these patients may be caused by confounding factors, such as mutation, chronological and biological age, cognitive status, motivation, and behavior [8]. The same observation may apply to respiratory muscle strength [33]. It may also be due to the diversity of DMD phenotypes, which Humbertclaude et al. showed in the French population; for example, the most severe group lost ambulation at a mean age of 7.10 years, as opposed to the mildest group, losing ambulation at 12.01 years old [34].

Perhaps the small size of our group size did not allow us to determine a relationship between MIP, MEP, HGS values, and genetic changes, or other confounding factors. However, taking into account our previous study, “E-monitoring of PULMonary function in patients with Duchenne Muscular Dystrophy at home,” (E-PULMoDMD; ClinicalTrials.gov Identifier: NCT05516745), was determined that daily regular spirometry measurements at home increase the quality and repeatability of the results obtained in the clinic and improve respiratory comfort, in some patients [35]. To improve the quality of the results obtained and to achieve greater repeatability, perhaps it should be recommended to perform more frequent measurements than those currently recommended (every 6–12 months) [36], especially since there are portable devices which can be used in homes [37,38].

Another interesting finding in our study was that all the individuals with DMD, regardless of the stage of the disease, presented low MIP, MEP, and HGS. Unlike this fact, most of the ambulatory patients were able to achieve maximum values, in the range of the results for healthy children, for FVC, FEV1, and PUL. From the results of the literature and our own research, it is known that the values of spirometry increase, reaching an individual maximum result before the loss of ambulation, while during the non-ambulatory period, these parameters are significantly decreased annually, regardless of the treatment with glucocorticosteroid [2,4,11,39,40]. PUL behaves similarly, and the study by Ricotti et al. showed that upper limb function (assessed with PUL) was stable in the ambulant population, while in the non-ambulant patients, an annual total loss of 4.13 points of PUL was observed [11]. 

However, in our study, we were surprised by the low values for muscle strength already obtained in the youngest children, with the function of gait still preserved. Finder et al. reported similar observations on MIP and MEP, based on the analysis of several studies [41].

Additionally, others point to the fact that MIP and MEP usually decrease faster than the values of spirometry, e.g., FVC and FEV1 in the natural history of the disease, which was already visible in children 6–7 years of age [40,42].

Our study showed that function indicators remained within the reference range for a healthy population, despite the already weakening strength of the respiratory muscles and the hand in ambulatory patients. While in the non-ambulatory group, there was a decrease in indicators of both the strength and function of the muscles of the evaluated organs. These results also partly explain the stronger correlation between upper limb condition and respiratory function for non-ambulant patients. This indicates the need for developing a specific care strategy that consists of respiratory function support and training, as well as taking care of the condition of the upper limbs.

In previous studies, other authors have evaluated indicators of strength and function regarding the hand and respiratory system to assess the course of the natural history of the disease, or to determine points in clinical trials. In our study, we draw attention to the possibility of using these dependencies in rehabilitation, thus improving the quality of life of patients with DMD.

In the current literature, there is a lack of guidelines according to upper limb physical therapy for DMD. The findings of this study highlight the meaning of upper limb rehabilitation in this population. The close anatomical relationship between the muscles of the upper limb and the respiratory muscles may cause that upper limb training can be considered as a respiratory muscle function-dependent activity [10]. Strengthening exercises of the upper limbs to support lung function might be considered potentially reasonable in a rehabilitation program for DMD patients. This strategy is successfully provided to patients with chronic obstructive pulmonary disease (COPD) or other lung diseases. In patients with COPD who additionally trained their upper body with resistance for four weeks, higher improvement in FEV1 and FVC was observed in comparison to the control group, which trained only with breathing exercises [43]. Moreover, other research shows that upper extremity muscle training increases inspiratory muscle strength in COPD patients [44].

Our previous study showed that it is possible to implement the telerehabilitation of respiratory muscles in patients with DMD [37,38]. After the results of the current research, we now consider the concept of the combined rehabilitation of both the respiratory system and the upper limbs.

The limitation of this study was that we did not explore the influence of skeletal disorders (scoliosis) on respiratory system function. The study was cross-sectional and lacked the evaluation of changes in the abovementioned parameters over time. As the number of participants was relatively low, our findings should be treated with caution. However, these observations justify addressing the question of whether upper limb exercises may contribute to the improvement of the pulmonary function in DMD patients, and may be indicators for further studies to determine the optimal strategy for preserving both parameters: upper limb strength and respiratory function. Future long-term studies may help to better understand the progression of upper limb weakness and respiratory muscle involvement.

## 5. Conclusions

1. The pulmonary and upper limb functions were within the normal range in ambulatory and lowered in non-ambulatory patients with DMD, but the muscle strength of both systems was low, regardless of the stage of the disease.
2. There seems to be an interdependence between the respiratory system and the upper limbs in terms of muscle strength and function in DMD patients, which is stronger in non-ambulatory patients. This may be the basis for the creation of a new personalized plan in rehabilitation—the simultaneous rehabilitation of the respiratory and upper limb muscles. Further studies on this topic should be conducted.

## 6. Clinical Implications

The rehabilitation of patients with DMD should focus on both the respiratory system and the upper limbs, preferably at the same time.

## Figures and Tables

**Table 1:** Patient characteristics and anthropometric data.

	All n = 53	Ambulant n = 28	Non-Ambulant n = 25
	Mean; SD/Median; IQR		
Age (years)	11.41; SD 3.70	9.17; SD 2.85	13.92; SD 2.87
Height (cm)	141.55; SD 19.09	130.16; SD 12.81	154.32; SD 13.86
Body weight (kg)	44.25; SD 19.24	34.44; SD 12.81	55.24; SD 19.48
BMI	21.17; SD 5.40	19.68; SD 4.15	22.84; SD 6.19
BMI z-score	0.55; SD 1.82	0.71; SD 1.07	0.38; SD 2.41
Age at the loss of ambulation (years)	10.41; 2.06	-	10.41; 2.06
**Mutation**			
Deletion	34 (64.2)	15 (53.6)	19 (76.0)
Duplication	8 (15.1)	5 (17.8)	3 (12.0)
Point mutation	5 (9.4)	4 (14.3)	1 (4.0)
Nonsense mutation	4 (7.5)	3 (10.7)	1(4.0)
No detected mutation	2 (3.8)	1 (3.6)	1 (4.0)
**Steroid therapy (ST)**	39 (%)	23 (%)	16 (%)
Encorton (prednisone) nb(%) Age at ST onset (year) Daily dose (mg) Calcort (deflazacort) nb(%) Age at ST onset (year) Daily dose (mg)	16 (41.0) 5.12, SD 1.08 24.37, SD 7.50 23 (58.9) 6.13, SD 2.37 15.80, SD 2.88	10 (43.4) 5.10; SD 1.28 11.2; SD 3.64 13 (56.6) 5.15; SD 2.44 14.07; SD 2.25	6 (37.5) 5.2; SD 1.48 12.42; SD4.79 10 (62.5) 7.4; SD 1.64 13.50; SD 6.04

BMI—body mass index.

**Table 2:** The values of respiratory muscle strength and spirometry.

Pulmonary Function Tests				
	All n = 53 Mean ± SD	Ambulant n = 28 Mean ± SD	Non-Ambulant n = 25 Mean ± SD	*p*-Value Ambulant vs. Non-Ambulant
	Respiratory muscle strength (cm H2O)			
**MIP** %pv	41.05 ±19.51 48.11 ± 27.38	45.57 ± 16.16 60.23 ± 24.10	36.00 ± 21.91 34.55 ± 24.65	0.127 <0.001 *
**MEP** %pv	40.05 ± 15.99 38.11 ± 22.77	44.67 ± 14.11 49.84 ± 22.23	34.88 ± 16.65 24.97 ± 15.06	0.024 * <0.001 *
Spirometry (Liters)				
**FVC** %pv	1.62 ± 0.54 77.32 ± 25.74	1.78 ±0.59 91.46 ± 14.01	1.61 ± 0.61 61.48 ± 26.84	0.209 0.001 *
**FEV1** %pv	1.81 ± 0.67 83.54 ± 28.39	1.83 ± 0.76 100.20 ± 16.60	1.63 ± 0.47 64.88 ± 27.38	0.204 0.013 *

FEV1—forced expiratory volume in 1 s; FVC—forced vital capacity; MIP—maximal inspiratory pressure; MEP—maximal expiratory pressure; * *p* < 0.05.

**Table 3:** The values of upper limb assessment.

Upper Limb				
	All n = 53 Mean ± SD	Ambulant n = 28 Mean ± SD	Non-Ambulant n = 25 Mean ± SD	*p*-Value Ambulant vs. Non-Ambulant
PUL %pv [score]	75.64 ± 27.86 31.37 ± 11.93	92.85 ± 9.76 39.00 ± 4.10	54.38 ± 28.77 22.84 1 ± 2.08	<0.001 <0.001
HGS %pv [kg]	33.28 ± 18.72 5.78 ± 3.31	43.35 ± 14.20 6.22 ± 2.53	21.52 ± 16.5 5.27 ± 4.04	<0.001 0.30

PUL—performance of upper limb; HGS—hand grip strength; kg—kilogram; pv—predicted value.

**Table 4:** Correlations between upper limb muscle function and respiratory parameters (%pv) (Spearman correlation test).

	All n = 53 R	*p*-Value	Ambulant n = 28 R	*p*-Value	Non-Ambulant n = 25 R	*p*-Value
Respiratory (%pv)	Upper Limb (%pv)					
	Hand grip strength					
**MIP**	0.61	<0.001 *	0.51	0.004 *	0.42	0.039 *
**MEP**	0.72	<0.001 *	0.52	0.004 *	0.74	<0.001 *
**FVC**	0.77	<0.001 *	0.47	0.001 *	0.81	<0.001 *
**FEV1**	0.79	<0.001 *	0.53	0.003 *	0.82	<0.001 *
	Performance of Upper Limb					
**MIP**	0.56	<0.001 *	0.29	0.133	0.43	0.028 *
**MEP**	0.69	<0.001 *	0.27	0.155	0.67	<0.001*
**FVC**	0.77	<0.001 *	0.41	0.028 *	0.84	<0.001 *
**FEV1**	0.77	<0.001 *	0.43	0.021 *	0.83	<0.001 *

FEV1—forced expiratory volume in 1 s; FVC—forced vital capacity, MIP—maximal inspiratory pressure; MEP—maximal expiratory pressure; PUL—performance of upper limb; pv—predicted value; * *p* < 0.05.

**Table 5:** Potential variables associated with hand grip strength (the univariable regression analysis).

	R2 = 0.739	
	b *	*p*-Value
PUL	1.140	0.000 *
FVC	0.6410	0.000 *
Age	0.448	0.005 *
Ambulation	−0.202	0.076
Height	0.233	0.197
Weight	−0.002	0.986
BMI z-score	0.177	0.281

PUL—performance of upper limb; FVC—forced expiratory volume; BMI—body mass index; * *p* < 0.05.

## Data Availability

The data presented in this study are available on request from the corresponding author. Full data are not publicly available due to privacy restrictions.

## Appendix A

Formulas used to calculate the percentage of predicted values of pulmonary function parameters [19].

MEP% = MEP × 100/(7619 + (7806 × age) + (0.004 × height × weight)

MIP% = −MIP × 100/(−27,020 − (4132 × age) − (0.003 × height × weight)
